# Looking to the future: How can clinical trials be designed in partnership with our participants?

**DOI:** 10.1002/alz.085979

**Published:** 2025-01-09

**Authors:** Jonathan M Schott

**Affiliations:** ^1^ Dementia Research Centre, UCL Queen Square Institute of Neurology, London United Kingdom

## Abstract

The recent positive phase 3 clinical trials of new treatments and their licensing and roll‐out in the US and other countries represents a major turning point in Alzheimer’s disease research. As has been the case with many other diseases, e.g. multiple sclerosis and stroke, it is hoped that these successes will act as a catalyst, spurring the development not only of the next generation of amyloid immunotherapies but a range of other therapeutic approaches targeting different aspects of Alzheimer pathology, and different forms of dementia. How future trials are designed will likely have major impacts on their chances of success but also how they are deployed when licensed in clinical practice. It is of course vital that trials are designed to prioritise patient safety and use robust methodologies to produce valid results while maximising the possibility of gaining insights into mechanisms and any side‐effects. However, it is also imperative that protocols are acceptable to participants, and designed wherever possible to maximise compliance, improve accessibility, reduce cost and increase representativeness. Factors that need to be considered include those which may be dependent on the drug’s mode of action and likely effect, e.g. the length of the trial, the method of trial drug delivery, and any requirements for safety monitoring. Factors where there may be scope for change include, but are not limited to, the required number of trial visits, the length of assessments (e.g. including shorter MRI protocols for safety monitoring), the possibility of remote or at home as opposed to in‐hospital visits where possible, and simplifying methods for determining molecular pathology both as entry and potentially stopping criteria (e.g. if plasma can be used instead of CSF or molecular imaging). If we are to maximise the reach of our trials, ensure they are truly representative of all the people who might benefit, and increase recruitment rates, it is vital that we engage with study participants in the design of future studies, engage them as advocates for our research, and promote and actively involve individuals from diverse and under‐represented backgrounds.

